# Supplementary material for: Modeling glioblastoma heterogeneity as a dynamic network of cell states
Source: Mol Syst Biol. 2021 Sep 16;17(9):e10105. doi: 10.15252/msb.202010105 (PMC8444284; doi:10.15252/msb.202010105)
Supplement: Supplementary file 5 — Source Data for Figure 3 [file MSB-17-e10105-s001.zip › Figure3A_sourcedata/GSEA_3065/nosigdb_state5.GseaPreranked.1622718435914/DOROSZKO_U3180MG_CLUSTER_3.html]

Details for gene set DOROSZKO\_U3180MG\_CLUSTER\_3[GSEA]

|  || Dataset | state5 |
| Phenotype | NoPhenotypeAvailable |
| Upregulated in class | na\_neg |
| GeneSet | DOROSZKO\_U3180MG\_CLUSTER\_3 |
| Enrichment Score (ES) | -0.51937765 |
| Normalized Enrichment Score (NES) | -2.050781 |
| Nominal p-value | 0.0 |
| FDR q-value | 0.0 |
| FWER p-Value | 0.0 |
Table: GSEA Results Summary

  

Fig 1: Enrichment plot: DOROSZKO\_U3180MG\_CLUSTER\_3      
 Profile of the Running ES Score & Positions of GeneSet Members on the Rank Ordered List

  

| PROBE | GENE SYMBOL | GENE\_TITLE | RANK IN GENE LIST | RANK METRIC SCORE | RUNNING ES | CORE ENRICHMENT || 1 | PGRMC1 |  |  | 52 | 0.639 | 0.0008 | No |
| 2 | CALU |  |  | 78 | 0.563 | 0.0040 | No |
| 3 | HACD3 |  |  | 91 | 0.542 | 0.0084 | No |
| 4 | XRCC2 |  |  | 94 | 0.534 | 0.0140 | No |
| 5 | GGH |  |  | 184 | 0.453 | 0.0084 | No |
| 6 | EMP2 |  |  | 239 | 0.414 | 0.0066 | No |
| 7 | CD151 |  |  | 243 | 0.412 | 0.0107 | No |
| 8 | TMX1 |  |  | 255 | 0.406 | 0.0138 | No |
| 9 | HELLS |  |  | 280 | 0.393 | 0.0152 | No |
| 10 | KDELR2 |  |  | 391 | 0.345 | 0.0061 | No |
| 11 | IKBIP |  |  | 414 | 0.335 | 0.0071 | No |
| 12 | ABHD3 |  |  | 458 | 0.323 | 0.0056 | No |
| 13 | DEGS1 |  |  | 467 | 0.321 | 0.0081 | No |
| 14 | GINS4 |  |  | 492 | 0.314 | 0.0087 | No |
| 15 | CDCA7 |  |  | 495 | 0.313 | 0.0118 | No |
| 16 | P4HB |  |  | 514 | 0.306 | 0.0130 | No |
| 17 | BRCA1 |  |  | 522 | 0.305 | 0.0155 | No |
| 18 | FANCA |  |  | 559 | 0.295 | 0.0145 | No |
| 19 | KDELC2 |  |  | 631 | 0.279 | 0.0092 | No |
| 20 | TECR |  |  | 633 | 0.278 | 0.0121 | No |
| 21 | SPATA33 |  |  | 658 | 0.274 | 0.0122 | No |
| 22 | NFATC2IP |  |  | 784 | 0.244 | 0.0002 | No |
| 23 | ORC6 |  |  | 806 | 0.240 | 0.0003 | No |
| 24 | DDB2 |  |  | 833 | 0.235 | -0.0002 | No |
| 25 | FUCA2 |  |  | 862 | 0.230 | -0.0010 | No |
| 26 | ATAD5 |  |  | 979 | 0.213 | -0.0123 | No |
| 27 | ASPM |  |  | 980 | 0.213 | -0.0100 | No |
| 28 | TMEM97 |  |  | 995 | 0.211 | -0.0094 | No |
| 29 | SLC20A1 |  |  | 1035 | 0.206 | -0.0117 | No |
| 30 | PRKDC |  |  | 1051 | 0.204 | -0.0113 | No |
| 31 | C21orf58 |  |  | 1066 | 0.203 | -0.0107 | No |
| 32 | TMEM107 |  |  | 1108 | 0.198 | -0.0134 | No |
| 33 | MMS22L |  |  | 1126 | 0.195 | -0.0133 | No |
| 34 | FADS1 |  |  | 1148 | 0.191 | -0.0136 | No |
| 35 | RBBP4 |  |  | 1218 | 0.183 | -0.0198 | No |
| 36 | CEP152 |  |  | 1436 | 0.159 | -0.0435 | No |
| 37 | CENPF |  |  | 1531 | 0.149 | -0.0530 | No |
| 38 | PAQR4 |  |  | 1643 | 0.138 | -0.0645 | No |
| 39 | RBL1 |  |  | 1669 | 0.136 | -0.0660 | No |
| 40 | FAM161A |  |  | 1697 | 0.133 | -0.0677 | No |
| 41 | BRIP1 |  |  | 1764 | 0.129 | -0.0740 | No |
| 42 | MIS18BP1 |  |  | 1765 | 0.129 | -0.0726 | No |
| 43 | BRCA2 |  |  | 1781 | 0.128 | -0.0730 | No |
| 44 | PRIM2 |  |  | 1881 | 0.120 | -0.0833 | No |
| 45 | CISD2 |  |  | 1902 | 0.118 | -0.0844 | No |
| 46 | SLF1 |  |  | 1925 | 0.117 | -0.0857 | No |
| 47 | PKMYT1 |  |  | 1940 | 0.116 | -0.0861 | No |
| 48 | PIDD1 |  |  | 1948 | 0.115 | -0.0857 | No |
| 49 | CENPJ |  |  | 2032 | 0.109 | -0.0942 | No |
| 50 | MCM8 |  |  | 2140 | 0.103 | -0.1057 | No |
| 51 | SCARA3 |  |  | 2149 | 0.102 | -0.1055 | No |
| 52 | MXD3 |  |  | 2178 | 0.100 | -0.1077 | No |
| 53 | CEP135 |  |  | 2201 | 0.098 | -0.1093 | No |
| 54 | NUP85 |  |  | 2246 | 0.095 | -0.1134 | No |
| 55 | DIAPH3 |  |  | 2387 | 0.086 | -0.1289 | No |
| 56 | MELK |  |  | 2402 | 0.085 | -0.1296 | No |
| 57 | SAAL1 |  |  | 2421 | 0.084 | -0.1308 | No |
| 58 | HAT1 |  |  | 2459 | 0.082 | -0.1343 | No |
| 59 | PHGDH |  |  | 2485 | 0.081 | -0.1363 | No |
| 60 | ATAD2 |  |  | 2545 | 0.077 | -0.1424 | No |
| 61 | KLHL23 |  |  | 2595 | 0.074 | -0.1474 | No |
| 62 | TMEM237 |  |  | 2680 | 0.069 | -0.1565 | No |
| 63 | MNS1 |  |  | 2792 | 0.063 | -0.1689 | No |
| 64 | SCCPDH |  |  | 2799 | 0.063 | -0.1689 | No |
| 65 | FAM111A |  |  | 2820 | 0.062 | -0.1706 | No |
| 66 | RAD18 |  |  | 2825 | 0.061 | -0.1704 | No |
| 67 | TEDC2 |  |  | 2834 | 0.061 | -0.1706 | No |
| 68 | FDPS |  |  | 2849 | 0.060 | -0.1716 | No |
| 69 | ARL6IP6 |  |  | 2850 | 0.060 | -0.1710 | No |
| 70 | AURKB |  |  | 2853 | 0.060 | -0.1706 | No |
| 71 | RTKN2 |  |  | 2869 | 0.059 | -0.1717 | No |
| 72 | CHEK1 |  |  | 2877 | 0.059 | -0.1719 | No |
| 73 | RAD51AP1 |  |  | 2905 | 0.057 | -0.1744 | No |
| 74 | CKAP2L |  |  | 2979 | 0.053 | -0.1824 | No |
| 75 | NRM |  |  | 3011 | 0.051 | -0.1855 | No |
| 76 | CASP8AP2 |  |  | 3046 | 0.050 | -0.1890 | No |
| 77 | RECQL4 |  |  | 3050 | 0.050 | -0.1888 | No |
| 78 | PIGX |  |  | 3058 | 0.049 | -0.1891 | No |
| 79 | SGO1 |  |  | 3060 | 0.049 | -0.1886 | No |
| 80 | MASTL |  |  | 3090 | 0.048 | -0.1915 | No |
| 81 | FANCD2 |  |  | 3151 | 0.044 | -0.1981 | No |
| 82 | LIG1 |  |  | 3181 | 0.042 | -0.2011 | No |
| 83 | NCAPG |  |  | 3194 | 0.041 | -0.2020 | No |
| 84 | CCP110 |  |  | 3202 | 0.041 | -0.2024 | No |
| 85 | RFC4 |  |  | 3212 | 0.041 | -0.2030 | No |
| 86 | PAXX |  |  | 3263 | 0.038 | -0.2085 | No |
| 87 | FOXM1 |  |  | 3279 | 0.038 | -0.2098 | No |
| 88 | CTPS1 |  |  | 3314 | 0.036 | -0.2134 | No |
| 89 | BUB1B |  |  | 3355 | 0.034 | -0.2178 | No |
| 90 | C19orf48 |  |  | 3373 | 0.033 | -0.2194 | No |
| 91 | SMC2 |  |  | 3374 | 0.032 | -0.2191 | No |
| 92 | FANCI |  |  | 3375 | 0.032 | -0.2187 | No |
| 93 | CDC6 |  |  | 3392 | 0.032 | -0.2202 | No |
| 94 | DEPDC1B |  |  | 3555 | 0.025 | -0.2390 | No |
| 95 | OLFM1 |  |  | 3559 | 0.025 | -0.2391 | No |
| 96 | FBXO4 |  |  | 3586 | 0.024 | -0.2419 | No |
| 97 | CNPY3 |  |  | 3592 | 0.024 | -0.2422 | No |
| 98 | WDR54 |  |  | 3602 | 0.023 | -0.2430 | No |
| 99 | KIFC1 |  |  | 3667 | 0.021 | -0.2503 | No |
| 100 | LSM8 |  |  | 3669 | 0.021 | -0.2502 | No |
| 101 | ESCO2 |  |  | 3672 | 0.021 | -0.2502 | No |
| 102 | SMC1A |  |  | 3696 | 0.020 | -0.2527 | No |
| 103 | PRIM1 |  |  | 3702 | 0.020 | -0.2530 | No |
| 104 | CIP2A |  |  | 3713 | 0.020 | -0.2540 | No |
| 105 | CEP57 |  |  | 3725 | 0.019 | -0.2551 | No |
| 106 | SPC24 |  |  | 3726 | 0.019 | -0.2549 | No |
| 107 | IER3IP1 |  |  | 3731 | 0.019 | -0.2551 | No |
| 108 | MSH6 |  |  | 3736 | 0.019 | -0.2554 | No |
| 109 | GTSE1 |  |  | 3738 | 0.018 | -0.2553 | No |
| 110 | PARPBP |  |  | 3820 | 0.015 | -0.2647 | No |
| 111 | ITGB3BP |  |  | 3830 | 0.014 | -0.2656 | No |
| 112 | PARP2 |  |  | 3831 | 0.014 | -0.2654 | No |
| 113 | CDC7 |  |  | 3855 | 0.014 | -0.2680 | No |
| 114 | RFC3 |  |  | 3863 | 0.013 | -0.2686 | No |
| 115 | MRPS11 |  |  | 3882 | 0.013 | -0.2706 | No |
| 116 | PANK2 |  |  | 3896 | 0.012 | -0.2720 | No |
| 117 | KNL1 |  |  | 3943 | 0.009 | -0.2773 | No |
| 118 | KIF15 |  |  | 3950 | 0.009 | -0.2779 | No |
| 119 | IMMT |  |  | 3978 | 0.008 | -0.2810 | No |
| 120 | NCAPD3 |  |  | 3996 | 0.007 | -0.2829 | No |
| 121 | NDC80 |  |  | 4034 | 0.006 | -0.2872 | No |
| 122 | NCAPH2 |  |  | 4124 | 0.002 | -0.2976 | No |
| 123 | TMUB1 |  |  | 4136 | 0.002 | -0.2989 | No |
| 124 | CCNE2 |  |  | 4180 | -0.001 | -0.3039 | No |
| 125 | NR2C2AP |  |  | 4217 | -0.002 | -0.3082 | No |
| 126 | GINS1 |  |  | 4224 | -0.002 | -0.3088 | No |
| 127 | WDHD1 |  |  | 4234 | -0.003 | -0.3099 | No |
| 128 | PRC1 |  |  | 4241 | -0.003 | -0.3105 | No |
| 129 | SRPK1 |  |  | 4294 | -0.006 | -0.3166 | No |
| 130 | SKA1 |  |  | 4308 | -0.006 | -0.3180 | No |
| 131 | FANCG |  |  | 4332 | -0.007 | -0.3207 | No |
| 132 | ASRGL1 |  |  | 4449 | -0.012 | -0.3341 | No |
| 133 | TACC3 |  |  | 4450 | -0.012 | -0.3340 | No |
| 134 | DONSON |  |  | 4454 | -0.012 | -0.3342 | No |
| 135 | CDK2 |  |  | 4489 | -0.013 | -0.3381 | No |
| 136 | HJURP |  |  | 4497 | -0.014 | -0.3388 | No |
| 137 | RBBP7 |  |  | 4521 | -0.015 | -0.3413 | No |
| 138 | HAUS2 |  |  | 4525 | -0.015 | -0.3415 | No |
| 139 | MCM4 |  |  | 4534 | -0.015 | -0.3423 | No |
| 140 | MCM10 |  |  | 4542 | -0.016 | -0.3429 | No |
| 141 | CDC45 |  |  | 4563 | -0.017 | -0.3451 | No |
| 142 | KIF2C |  |  | 4621 | -0.019 | -0.3516 | No |
| 143 | NUF2 |  |  | 4628 | -0.019 | -0.3521 | No |
| 144 | TCF19 |  |  | 4635 | -0.020 | -0.3525 | No |
| 145 | CENPK |  |  | 4675 | -0.021 | -0.3569 | No |
| 146 | SGO2 |  |  | 4709 | -0.022 | -0.3605 | No |
| 147 | WDR76 |  |  | 4723 | -0.023 | -0.3618 | No |
| 148 | CDK1 |  |  | 4766 | -0.025 | -0.3665 | No |
| 149 | BARD1 |  |  | 4776 | -0.025 | -0.3673 | No |
| 150 | FAM111B |  |  | 4792 | -0.025 | -0.3688 | No |
| 151 | MCM2 |  |  | 4824 | -0.027 | -0.3721 | No |
| 152 | VKORC1 |  |  | 4834 | -0.027 | -0.3729 | No |
| 153 | SHCBP1 |  |  | 4848 | -0.027 | -0.3741 | No |
| 154 | LRRC45 |  |  | 4851 | -0.027 | -0.3740 | No |
| 155 | EZH2 |  |  | 4858 | -0.028 | -0.3744 | No |
| 156 | FAF1 |  |  | 4893 | -0.029 | -0.3781 | No |
| 157 | DTL |  |  | 4909 | -0.029 | -0.3796 | No |
| 158 | MTHFD1 |  |  | 4914 | -0.030 | -0.3797 | No |
| 159 | DPM2 |  |  | 4926 | -0.030 | -0.3807 | No |
| 160 | NUDT15 |  |  | 4932 | -0.030 | -0.3809 | No |
| 161 | DSCC1 |  |  | 4941 | -0.031 | -0.3815 | No |
| 162 | DDX39A |  |  | 4948 | -0.031 | -0.3819 | No |
| 163 | MCM7 |  |  | 4986 | -0.032 | -0.3859 | No |
| 164 | CHAC2 |  |  | 4987 | -0.032 | -0.3855 | No |
| 165 | GINS2 |  |  | 5024 | -0.034 | -0.3894 | No |
| 166 | HAUS8 |  |  | 5039 | -0.034 | -0.3907 | No |
| 167 | RCC1 |  |  | 5076 | -0.036 | -0.3945 | No |
| 168 | CDCA5 |  |  | 5167 | -0.039 | -0.4047 | No |
| 169 | CDT1 |  |  | 5191 | -0.040 | -0.4069 | No |
| 170 | TEDC1 |  |  | 5198 | -0.040 | -0.4072 | No |
| 171 | PSMC3IP |  |  | 5222 | -0.041 | -0.4095 | No |
| 172 | POLA2 |  |  | 5229 | -0.042 | -0.4097 | No |
| 173 | NCAPH |  |  | 5287 | -0.044 | -0.4159 | No |
| 174 | FARSB |  |  | 5293 | -0.044 | -0.4160 | No |
| 175 | NCAPG2 |  |  | 5309 | -0.045 | -0.4173 | No |
| 176 | SPC25 |  |  | 5312 | -0.045 | -0.4171 | No |
| 177 | UHRF1 |  |  | 5337 | -0.046 | -0.4194 | No |
| 178 | NUDT21 |  |  | 5349 | -0.047 | -0.4202 | No |
| 179 | NUSAP1 |  |  | 5389 | -0.048 | -0.4242 | No |
| 180 | POLD3 |  |  | 5393 | -0.048 | -0.4241 | No |
| 181 | HIRIP3 |  |  | 5440 | -0.050 | -0.4289 | No |
| 182 | ACYP1 |  |  | 5479 | -0.052 | -0.4328 | No |
| 183 | MACROD1 |  |  | 5525 | -0.054 | -0.4375 | No |
| 184 | SMC3 |  |  | 5536 | -0.054 | -0.4381 | No |
| 185 | TRIP13 |  |  | 5570 | -0.056 | -0.4414 | No |
| 186 | BUB3 |  |  | 5596 | -0.057 | -0.4437 | No |
| 187 | CLSPN |  |  | 5614 | -0.058 | -0.4451 | No |
| 188 | MSH2 |  |  | 5621 | -0.059 | -0.4451 | No |
| 189 | CCNA2 |  |  | 5671 | -0.061 | -0.4502 | No |
| 190 | ARRB2 |  |  | 5685 | -0.062 | -0.4511 | No |
| 191 | TIMELESS |  |  | 5717 | -0.063 | -0.4540 | No |
| 192 | TOP2A |  |  | 5722 | -0.063 | -0.4538 | No |
| 193 | CDCA3 |  |  | 5726 | -0.063 | -0.4535 | No |
| 194 | COA1 |  |  | 5751 | -0.064 | -0.4556 | No |
| 195 | KIF22 |  |  | 5758 | -0.064 | -0.4556 | No |
| 196 | RMI2 |  |  | 5797 | -0.065 | -0.4594 | No |
| 197 | KPNA2 |  |  | 5811 | -0.066 | -0.4602 | No |
| 198 | MGME1 |  |  | 5818 | -0.066 | -0.4602 | No |
| 199 | LY6E |  |  | 5890 | -0.069 | -0.4678 | No |
| 200 | RACGAP1 |  |  | 5902 | -0.070 | -0.4683 | No |
| 201 | CDCA4 |  |  | 5928 | -0.071 | -0.4705 | No |
| 202 | DSN1 |  |  | 5931 | -0.071 | -0.4700 | No |
| 203 | RNASEH2B |  |  | 5954 | -0.072 | -0.4718 | No |
| 204 | CENPQ |  |  | 5972 | -0.073 | -0.4730 | No |
| 205 | PRTFDC1 |  |  | 5982 | -0.073 | -0.4732 | No |
| 206 | COMMD4 |  |  | 6015 | -0.075 | -0.4762 | No |
| 207 | EXOSC8 |  |  | 6020 | -0.075 | -0.4758 | No |
| 208 | MCMBP |  |  | 6026 | -0.076 | -0.4756 | No |
| 209 | LRR1 |  |  | 6149 | -0.082 | -0.4890 | No |
| 210 | CHAF1A |  |  | 6157 | -0.082 | -0.4890 | No |
| 211 | CSE1L |  |  | 6160 | -0.083 | -0.4883 | No |
| 212 | RFC5 |  |  | 6186 | -0.084 | -0.4903 | No |
| 213 | MKI67 |  |  | 6211 | -0.086 | -0.4922 | No |
| 214 | XRCC5 |  |  | 6246 | -0.087 | -0.4953 | No |
| 215 | RAC3 |  |  | 6266 | -0.088 | -0.4965 | No |
| 216 | PSMD14 |  |  | 6275 | -0.089 | -0.4965 | No |
| 217 | INAFM1 |  |  | 6284 | -0.090 | -0.4965 | No |
| 218 | RAB23 |  |  | 6294 | -0.090 | -0.4966 | No |
| 219 | RRM1 |  |  | 6298 | -0.090 | -0.4959 | No |
| 220 | RRM2 |  |  | 6308 | -0.091 | -0.4960 | No |
| 221 | CENPM |  |  | 6368 | -0.093 | -0.5019 | No |
| 222 | CKAP2 |  |  | 6372 | -0.094 | -0.5013 | No |
| 223 | ASF1B |  |  | 6400 | -0.095 | -0.5034 | No |
| 224 | POC1A |  |  | 6411 | -0.096 | -0.5035 | No |
| 225 | SNX5 |  |  | 6418 | -0.097 | -0.5032 | No |
| 226 | ARHGAP11A |  |  | 6423 | -0.097 | -0.5026 | No |
| 227 | ZWINT |  |  | 6453 | -0.099 | -0.5050 | No |
| 228 | DNMT1 |  |  | 6455 | -0.099 | -0.5040 | No |
| 229 | UNG |  |  | 6479 | -0.100 | -0.5056 | No |
| 230 | PRADC1 |  |  | 6503 | -0.101 | -0.5072 | No |
| 231 | SHMT2 |  |  | 6514 | -0.102 | -0.5073 | No |
| 232 | SMC4 |  |  | 6517 | -0.102 | -0.5064 | No |
| 233 | E2F1 |  |  | 6528 | -0.103 | -0.5065 | No |
| 234 | KIF4A |  |  | 6529 | -0.103 | -0.5053 | No |
| 235 | MCM5 |  |  | 6553 | -0.105 | -0.5069 | No |
| 236 | PIMREG |  |  | 6566 | -0.106 | -0.5072 | No |
| 237 | VOPP1 |  |  | 6570 | -0.106 | -0.5064 | No |
| 238 | DBF4 |  |  | 6572 | -0.106 | -0.5053 | No |
| 239 | NUDT5 |  |  | 6590 | -0.107 | -0.5062 | No |
| 240 | MAD2L1 |  |  | 6600 | -0.108 | -0.5060 | No |
| 241 | PPIF |  |  | 6616 | -0.109 | -0.5066 | No |
| 242 | XRN2 |  |  | 6620 | -0.109 | -0.5058 | No |
| 243 | DCK |  |  | 6637 | -0.110 | -0.5065 | No |
| 244 | LMNB1 |  |  | 6638 | -0.110 | -0.5053 | No |
| 245 | VRK1 |  |  | 6651 | -0.111 | -0.5055 | No |
| 246 | ANP32E |  |  | 6655 | -0.111 | -0.5046 | No |
| 247 | RPA2 |  |  | 6705 | -0.114 | -0.5092 | No |
| 248 | DEPDC1 |  |  | 6723 | -0.115 | -0.5099 | No |
| 249 | CDCA2 |  |  | 6730 | -0.116 | -0.5094 | No |
| 250 | PSMG2 |  |  | 6743 | -0.117 | -0.5095 | No |
| 251 | KIF23 |  |  | 6764 | -0.118 | -0.5106 | No |
| 252 | UBR7 |  |  | 6772 | -0.119 | -0.5101 | No |
| 253 | FBXO5 |  |  | 6775 | -0.119 | -0.5091 | No |
| 254 | RAD1 |  |  | 6786 | -0.120 | -0.5089 | No |
| 255 | HPF1 |  |  | 6811 | -0.122 | -0.5104 | No |
| 256 | ZWILCH |  |  | 6812 | -0.122 | -0.5091 | No |
| 257 | CCDC34 |  |  | 6826 | -0.123 | -0.5093 | No |
| 258 | PFKP |  |  | 6845 | -0.124 | -0.5101 | No |
| 259 | CCND3 |  |  | 6881 | -0.128 | -0.5128 | No |
| 260 | SUZ12 |  |  | 6889 | -0.128 | -0.5122 | No |
| 261 | NFYB |  |  | 6899 | -0.129 | -0.5119 | No |
| 262 | GNG4 |  |  | 6906 | -0.129 | -0.5112 | No |
| 263 | SF3B4 |  |  | 6929 | -0.131 | -0.5124 | No |
| 264 | MND1 |  |  | 6972 | -0.135 | -0.5158 | No |
| 265 | PLP2 |  |  | 6975 | -0.135 | -0.5146 | No |
| 266 | RAD51C |  |  | 6989 | -0.136 | -0.5147 | No |
| 267 | PBK |  |  | 7010 | -0.138 | -0.5155 | No |
| 268 | CCT2 |  |  | 7013 | -0.138 | -0.5143 | No |
| 269 | GPN3 |  |  | 7039 | -0.140 | -0.5157 | No |
| 270 | DNAJC9 |  |  | 7043 | -0.141 | -0.5145 | No |
| 271 | TMEM54 |  |  | 7074 | -0.143 | -0.5165 | No |
| 272 | RBBP8 |  |  | 7099 | -0.145 | -0.5177 | No |
| 273 | POLE3 |  |  | 7101 | -0.146 | -0.5162 | No |
| 274 | SVIP |  |  | 7114 | -0.146 | -0.5161 | No |
| 275 | MIS18A |  |  | 7115 | -0.147 | -0.5145 | No |
| 276 | CENPW |  |  | 7116 | -0.147 | -0.5129 | No |
| 277 | KPNB1 |  |  | 7144 | -0.150 | -0.5144 | No |
| 278 | TFDP1 |  |  | 7187 | -0.154 | -0.5177 | Yes |
| 279 | ACTL6A |  |  | 7196 | -0.155 | -0.5170 | Yes |
| 280 | FEN1 |  |  | 7201 | -0.155 | -0.5158 | Yes |
| 281 | JPT2 |  |  | 7212 | -0.157 | -0.5152 | Yes |
| 282 | LRRCC1 |  |  | 7223 | -0.158 | -0.5147 | Yes |
| 283 | GMNN |  |  | 7224 | -0.158 | -0.5130 | Yes |
| 284 | EEF1E1 |  |  | 7241 | -0.160 | -0.5131 | Yes |
| 285 | NDUFB11 |  |  | 7246 | -0.161 | -0.5118 | Yes |
| 286 | TRAP1 |  |  | 7267 | -0.163 | -0.5124 | Yes |
| 287 | AHCY |  |  | 7274 | -0.164 | -0.5113 | Yes |
| 288 | HSPB11 |  |  | 7286 | -0.166 | -0.5108 | Yes |
| 289 | TMPO |  |  | 7296 | -0.167 | -0.5101 | Yes |
| 290 | PMVK |  |  | 7311 | -0.168 | -0.5099 | Yes |
| 291 | SRR |  |  | 7319 | -0.169 | -0.5089 | Yes |
| 292 | SUPT16H |  |  | 7322 | -0.170 | -0.5073 | Yes |
| 293 | CENPH |  |  | 7329 | -0.171 | -0.5062 | Yes |
| 294 | MCM6 |  |  | 7375 | -0.178 | -0.5095 | Yes |
| 295 | TK1 |  |  | 7377 | -0.178 | -0.5077 | Yes |
| 296 | TCEAL9 |  |  | 7389 | -0.180 | -0.5071 | Yes |
| 297 | TUBB6 |  |  | 7396 | -0.180 | -0.5058 | Yes |
| 298 | RDX |  |  | 7397 | -0.181 | -0.5039 | Yes |
| 299 | USP1 |  |  | 7398 | -0.181 | -0.5019 | Yes |
| 300 | RPA1 |  |  | 7418 | -0.183 | -0.5021 | Yes |
| 301 | CBR3 |  |  | 7420 | -0.184 | -0.5003 | Yes |
| 302 | MRM2 |  |  | 7427 | -0.185 | -0.4990 | Yes |
| 303 | ENOPH1 |  |  | 7445 | -0.186 | -0.4990 | Yes |
| 304 | CMSS1 |  |  | 7453 | -0.187 | -0.4978 | Yes |
| 305 | PSIP1 |  |  | 7460 | -0.187 | -0.4964 | Yes |
| 306 | HNRNPA3 |  |  | 7470 | -0.188 | -0.4955 | Yes |
| 307 | CENPN |  |  | 7528 | -0.194 | -0.5000 | Yes |
| 308 | CMC2 |  |  | 7539 | -0.196 | -0.4991 | Yes |
| 309 | MAGOHB |  |  | 7542 | -0.196 | -0.4972 | Yes |
| 310 | CKLF |  |  | 7547 | -0.197 | -0.4955 | Yes |
| 311 | TPX2 |  |  | 7549 | -0.197 | -0.4935 | Yes |
| 312 | ACADM |  |  | 7551 | -0.198 | -0.4915 | Yes |
| 313 | MCM3 |  |  | 7552 | -0.198 | -0.4894 | Yes |
| 314 | UBE2T |  |  | 7561 | -0.199 | -0.4881 | Yes |
| 315 | SAE1 |  |  | 7570 | -0.200 | -0.4869 | Yes |
| 316 | TMEM14C |  |  | 7572 | -0.200 | -0.4849 | Yes |
| 317 | BIRC5 |  |  | 7596 | -0.204 | -0.4854 | Yes |
| 318 | BTG3 |  |  | 7602 | -0.204 | -0.4837 | Yes |
| 319 | PPIH |  |  | 7605 | -0.205 | -0.4818 | Yes |
| 320 | EIF4A3 |  |  | 7619 | -0.207 | -0.4810 | Yes |
| 321 | DERA |  |  | 7626 | -0.207 | -0.4795 | Yes |
| 322 | TEX30 |  |  | 7639 | -0.209 | -0.4786 | Yes |
| 323 | CARHSP1 |  |  | 7667 | -0.213 | -0.4795 | Yes |
| 324 | BAZ1B |  |  | 7669 | -0.213 | -0.4773 | Yes |
| 325 | AK2 |  |  | 7671 | -0.213 | -0.4751 | Yes |
| 326 | LMNB2 |  |  | 7725 | -0.222 | -0.4789 | Yes |
| 327 | PCLAF |  |  | 7734 | -0.223 | -0.4775 | Yes |
| 328 | HAUS1 |  |  | 7761 | -0.227 | -0.4781 | Yes |
| 329 | YEATS4 |  |  | 7764 | -0.227 | -0.4758 | Yes |
| 330 | RAD21 |  |  | 7785 | -0.231 | -0.4757 | Yes |
| 331 | MAPRE1 |  |  | 7808 | -0.236 | -0.4757 | Yes |
| 332 | SAC3D1 |  |  | 7825 | -0.240 | -0.4750 | Yes |
| 333 | PXMP2 |  |  | 7827 | -0.240 | -0.4725 | Yes |
| 334 | TCTEX1D2 |  |  | 7841 | -0.242 | -0.4714 | Yes |
| 335 | TUBG1 |  |  | 7846 | -0.243 | -0.4693 | Yes |
| 336 | NRGN |  |  | 7858 | -0.245 | -0.4679 | Yes |
| 337 | WDR34 |  |  | 7862 | -0.246 | -0.4656 | Yes |
| 338 | HDAC2 |  |  | 7865 | -0.247 | -0.4631 | Yes |
| 339 | CENPU |  |  | 7871 | -0.248 | -0.4610 | Yes |
| 340 | DHFR |  |  | 7878 | -0.250 | -0.4590 | Yes |
| 341 | PSMG1 |  |  | 7880 | -0.250 | -0.4564 | Yes |
| 342 | COPS3 |  |  | 7912 | -0.258 | -0.4573 | Yes |
| 343 | ARPC1B |  |  | 7917 | -0.259 | -0.4550 | Yes |
| 344 | TIMM10 |  |  | 7925 | -0.261 | -0.4530 | Yes |
| 345 | PSMB9 |  |  | 7929 | -0.262 | -0.4505 | Yes |
| 346 | MRPL37 |  |  | 7946 | -0.265 | -0.4495 | Yes |
| 347 | VPS29 |  |  | 7960 | -0.268 | -0.4481 | Yes |
| 348 | H2AFX |  |  | 7965 | -0.269 | -0.4457 | Yes |
| 349 | CKS2 |  |  | 7972 | -0.270 | -0.4434 | Yes |
| 350 | HMGB2 |  |  | 7973 | -0.271 | -0.4405 | Yes |
| 351 | MAD2L2 |  |  | 7998 | -0.275 | -0.4403 | Yes |
| 352 | CENPV |  |  | 8000 | -0.276 | -0.4375 | Yes |
| 353 | PSMA3 |  |  | 8006 | -0.277 | -0.4351 | Yes |
| 354 | MCUB |  |  | 8009 | -0.277 | -0.4323 | Yes |
| 355 | MYBL2 |  |  | 8041 | -0.283 | -0.4329 | Yes |
| 356 | RFC2 |  |  | 8049 | -0.285 | -0.4306 | Yes |
| 357 | CHCHD3 |  |  | 8055 | -0.287 | -0.4281 | Yes |
| 358 | UBA2 |  |  | 8067 | -0.290 | -0.4262 | Yes |
| 359 | CENPX |  |  | 8069 | -0.290 | -0.4232 | Yes |
| 360 | TPRKB |  |  | 8072 | -0.291 | -0.4203 | Yes |
| 361 | DTYMK |  |  | 8075 | -0.292 | -0.4174 | Yes |
| 362 | CTNNAL1 |  |  | 8076 | -0.292 | -0.4142 | Yes |
| 363 | CDKN3 |  |  | 8079 | -0.293 | -0.4113 | Yes |
| 364 | PCNA |  |  | 8082 | -0.293 | -0.4083 | Yes |
| 365 | PHF19 |  |  | 8093 | -0.295 | -0.4063 | Yes |
| 366 | ECI1 |  |  | 8099 | -0.297 | -0.4037 | Yes |
| 367 | NABP2 |  |  | 8111 | -0.299 | -0.4017 | Yes |
| 368 | DUT |  |  | 8118 | -0.300 | -0.3992 | Yes |
| 369 | CCT5 |  |  | 8125 | -0.302 | -0.3966 | Yes |
| 370 | MZT2A |  |  | 8135 | -0.305 | -0.3943 | Yes |
| 371 | TMEM106C |  |  | 8146 | -0.308 | -0.3922 | Yes |
| 372 | MCUR1 |  |  | 8150 | -0.309 | -0.3892 | Yes |
| 373 | ATP5MC1 |  |  | 8169 | -0.317 | -0.3879 | Yes |
| 374 | CBX5 |  |  | 8171 | -0.318 | -0.3845 | Yes |
| 375 | FKBP3 |  |  | 8175 | -0.319 | -0.3814 | Yes |
| 376 | HIST1H1E |  |  | 8176 | -0.320 | -0.3780 | Yes |
| 377 | SSRP1 |  |  | 8178 | -0.320 | -0.3746 | Yes |
| 378 | PARP1 |  |  | 8182 | -0.321 | -0.3715 | Yes |
| 379 | H2AFY |  |  | 8189 | -0.323 | -0.3687 | Yes |
| 380 | PPP1CA |  |  | 8193 | -0.324 | -0.3656 | Yes |
| 381 | CCT4 |  |  | 8211 | -0.329 | -0.3640 | Yes |
| 382 | CKS1B |  |  | 8241 | -0.336 | -0.3637 | Yes |
| 383 | SUMO3 |  |  | 8244 | -0.337 | -0.3603 | Yes |
| 384 | KRBOX1 |  |  | 8249 | -0.339 | -0.3571 | Yes |
| 385 | CCDC167 |  |  | 8260 | -0.342 | -0.3546 | Yes |
| 386 | PSMC3 |  |  | 8261 | -0.342 | -0.3509 | Yes |
| 387 | AKR7A2 |  |  | 8264 | -0.343 | -0.3474 | Yes |
| 388 | UBE2C |  |  | 8278 | -0.348 | -0.3452 | Yes |
| 389 | MRPS26 |  |  | 8285 | -0.350 | -0.3421 | Yes |
| 390 | NFIC |  |  | 8297 | -0.354 | -0.3395 | Yes |
| 391 | CSNK2B |  |  | 8298 | -0.354 | -0.3357 | Yes |
| 392 | DCTPP1 |  |  | 8303 | -0.356 | -0.3323 | Yes |
| 393 | EIF4EBP1 |  |  | 8306 | -0.356 | -0.3287 | Yes |
| 394 | HNRNPF |  |  | 8319 | -0.360 | -0.3262 | Yes |
| 395 | PA2G4 |  |  | 8320 | -0.360 | -0.3223 | Yes |
| 396 | XRCC6 |  |  | 8325 | -0.361 | -0.3189 | Yes |
| 397 | GLO1 |  |  | 8338 | -0.364 | -0.3163 | Yes |
| 398 | OLA1 |  |  | 8341 | -0.367 | -0.3126 | Yes |
| 399 | TUBB4B |  |  | 8356 | -0.371 | -0.3102 | Yes |
| 400 | MRPL18 |  |  | 8358 | -0.373 | -0.3063 | Yes |
| 401 | NUDT1 |  |  | 8359 | -0.373 | -0.3022 | Yes |
| 402 | CLIC1 |  |  | 8368 | -0.378 | -0.2991 | Yes |
| 403 | ARPC5L |  |  | 8371 | -0.379 | -0.2952 | Yes |
| 404 | SNRPF |  |  | 8382 | -0.382 | -0.2923 | Yes |
| 405 | TALDO1 |  |  | 8393 | -0.386 | -0.2892 | Yes |
| 406 | SLBP |  |  | 8396 | -0.387 | -0.2853 | Yes |
| 407 | GTF3A |  |  | 8399 | -0.388 | -0.2813 | Yes |
| 408 | RALY |  |  | 8402 | -0.388 | -0.2773 | Yes |
| 409 | SNRPD3 |  |  | 8423 | -0.395 | -0.2754 | Yes |
| 410 | C3orf14 |  |  | 8424 | -0.396 | -0.2711 | Yes |
| 411 | HMGB3 |  |  | 8427 | -0.399 | -0.2670 | Yes |
| 412 | HNRNPD |  |  | 8436 | -0.402 | -0.2636 | Yes |
| 413 | ACAT2 |  |  | 8440 | -0.404 | -0.2596 | Yes |
| 414 | HNRNPAB |  |  | 8441 | -0.404 | -0.2552 | Yes |
| 415 | NUDC |  |  | 8442 | -0.404 | -0.2509 | Yes |
| 416 | GAMT |  |  | 8449 | -0.407 | -0.2472 | Yes |
| 417 | PGK1 |  |  | 8452 | -0.408 | -0.2430 | Yes |
| 418 | ITGB1BP1 |  |  | 8459 | -0.413 | -0.2392 | Yes |
| 419 | H1FX |  |  | 8466 | -0.417 | -0.2354 | Yes |
| 420 | CACYBP |  |  | 8486 | -0.428 | -0.2330 | Yes |
| 421 | GGCT |  |  | 8493 | -0.432 | -0.2290 | Yes |
| 422 | SNRNP25 |  |  | 8494 | -0.432 | -0.2243 | Yes |
| 423 | IDH2 |  |  | 8498 | -0.434 | -0.2200 | Yes |
| 424 | HIST1H1D |  |  | 8520 | -0.443 | -0.2176 | Yes |
| 425 | MRPL17 |  |  | 8528 | -0.446 | -0.2136 | Yes |
| 426 | PAFAH1B3 |  |  | 8532 | -0.448 | -0.2091 | Yes |
| 427 | ALYREF |  |  | 8535 | -0.449 | -0.2045 | Yes |
| 428 | POLE4 |  |  | 8536 | -0.451 | -0.1996 | Yes |
| 429 | SNRPD1 |  |  | 8537 | -0.451 | -0.1947 | Yes |
| 430 | EEF1B2 |  |  | 8557 | -0.463 | -0.1919 | Yes |
| 431 | UQCC2 |  |  | 8562 | -0.465 | -0.1874 | Yes |
| 432 | RPA3 |  |  | 8572 | -0.471 | -0.1833 | Yes |
| 433 | NME1 |  |  | 8573 | -0.471 | -0.1782 | Yes |
| 434 | MINOS1 |  |  | 8582 | -0.476 | -0.1740 | Yes |
| 435 | TYMS |  |  | 8599 | -0.488 | -0.1706 | Yes |
| 436 | POP7 |  |  | 8605 | -0.495 | -0.1658 | Yes |
| 437 | ETFB |  |  | 8610 | -0.499 | -0.1609 | Yes |
| 438 | PIN1 |  |  | 8623 | -0.509 | -0.1568 | Yes |
| 439 | CDK4 |  |  | 8625 | -0.510 | -0.1514 | Yes |
| 440 | PEG10 |  |  | 8628 | -0.510 | -0.1461 | Yes |
| 441 | METRN |  |  | 8638 | -0.518 | -0.1415 | Yes |
| 442 | POLR2I |  |  | 8641 | -0.519 | -0.1362 | Yes |
| 443 | TUBA1C |  |  | 8648 | -0.524 | -0.1312 | Yes |
| 444 | SIVA1 |  |  | 8654 | -0.529 | -0.1260 | Yes |
| 445 | MEA1 |  |  | 8661 | -0.533 | -0.1210 | Yes |
| 446 | BCL7C |  |  | 8666 | -0.536 | -0.1156 | Yes |
| 447 | HMGA1 |  |  | 8675 | -0.543 | -0.1107 | Yes |
| 448 | ANP32B |  |  | 8680 | -0.545 | -0.1052 | Yes |
| 449 | SNRPB |  |  | 8683 | -0.547 | -0.0996 | Yes |
| 450 | NDUFA6 |  |  | 8687 | -0.550 | -0.0940 | Yes |
| 451 | H2AFV |  |  | 8693 | -0.554 | -0.0885 | Yes |
| 452 | PGAM1 |  |  | 8698 | -0.558 | -0.0830 | Yes |
| 453 | BANF1 |  |  | 8707 | -0.567 | -0.0778 | Yes |
| 454 | PDCD5 |  |  | 8729 | -0.592 | -0.0738 | Yes |
| 455 | YWHAQ |  |  | 8730 | -0.592 | -0.0674 | Yes |
| 456 | TUBB |  |  | 8763 | -0.621 | -0.0644 | Yes |
| 457 | DEK |  |  | 8770 | -0.627 | -0.0583 | Yes |
| 458 | H2AFZ |  |  | 8774 | -0.630 | -0.0519 | Yes |
| 459 | COX5A |  |  | 8785 | -0.639 | -0.0461 | Yes |
| 460 | AP2S1 |  |  | 8799 | -0.648 | -0.0406 | Yes |
| 461 | LSM3 |  |  | 8803 | -0.655 | -0.0339 | Yes |
| 462 | MZT2B |  |  | 8816 | -0.665 | -0.0281 | Yes |
| 463 | RANBP1 |  |  | 8819 | -0.669 | -0.0211 | Yes |
| 464 | NME4 |  |  | 8824 | -0.678 | -0.0142 | Yes |
| 465 | SMS |  |  | 8834 | -0.690 | -0.0078 | Yes |
| 466 | HIST1H4C |  |  | 8850 | -0.710 | -0.0019 | Yes |
| 467 | LSM4 |  |  | 8853 | -0.712 | 0.0056 | Yes |
| 468 | CSRP2 |  |  | 8954 | -0.891 | 0.0035 | Yes |
Table: GSEA details [plain text format]

  

Fig 2: DOROSZKO\_U3180MG\_CLUSTER\_3: Random ES distribution      
 Gene set null distribution of ES for **DOROSZKO\_U3180MG\_CLUSTER\_3**
